# *N*-Substituted Benzyl Matrinic Acid Derivatives Inhibit Hepatitis C Virus (HCV) Replication through Down-Regulating Host Heat-Stress Cognate 70 (Hsc70) Expression

**DOI:** 10.1371/journal.pone.0058675

**Published:** 2013-03-14

**Authors:** Na-Na Du, Zong-Gen Peng, Chong-Wen Bi, Sheng Tang, Ying-Hong Li, Jian-Rui Li, Yan-Ping Zhu, Jing-Pu Zhang, Yan-Xiang Wang, Jian-Dong Jiang, Dan-Qing Song

**Affiliations:** 1 Institute of Medicinal Biotechnology, Chinese Academy of Medical Science and Peking Union Medical College, Beijing, China; 2 State Key Laboratory of Bioactive Substance and Functions of Natural Medicines, Institute of Materia Medica, Chinese Academy of Medical Sciences and Peking Union Medical College, Beijing, China; Kobe University, Japan

## Abstract

Heat-stress cognate 70 (Hsc70) is a host factor that helps hepatitis C virus (HCV) to complete its life cycle in infected hepatocytes. Using Hsc70 as a target for HCV inhibition, a series of novel *N*-substituted benzyl matrinic/sophoridinic acid derivatives was synthesized and evaluated for their anti-HCV activity in vitro. Among these analogues, compound **7c** possessing *N*-*p*-methylbenzyl afforded an appealing ability to inhibit HCV replication with SI value over 53. Furthermore, it showed a good oral pharmacokinetic profile with area-under-curve (AUC) of 13.4 µM·h, and a considerably good safety in oral administration in mice (LD_50_>1000 mg/kg). As **7c** suppresses HCV replication via an action mode distinctly different from that of the marketed anti-HCV drugs, it has been selected as a new mechanism anti-HCV candidate for further investigation, with an advantage of no or decreased chance to induce drug-resistant mutations.

## Introduction

Currently, hepatitis C virus (HCV) infection is a significant health problem worldwide. Standard therapy for HCV infection in clinic is the combination of pegylated-interferon with ribavirin [1]. However, this treatment regimen is only effective in about 40%−50% of patients infected with HCV genotype-1, which accounts for the majority of infections in the USA, Europe and Asia [2], [3]. Meanwhile, serious adverse effects such as depression and flu-like symptoms also limit its application [3], [4]. Two small molecular inhibitors for HCV nonstructural protein 3/4A (NS3/4A) protease, telaprevir and boceprevir, were approved by the Food and Drug Administration (FDA) in 2011 [5]–[7]. The NS3/4A inhibitors provide more therapeutic options for clinicians. However, antiviral therapy targeting specific viral enzyme such as HCV protease causes the emergence of drug-resistant mutations [8]–[11]. New anti-HCV therapeutic drugs with novel mechanisms that causes no or decreased chance of inducing drug-resistance are highly desirable.

**Figure 1:**
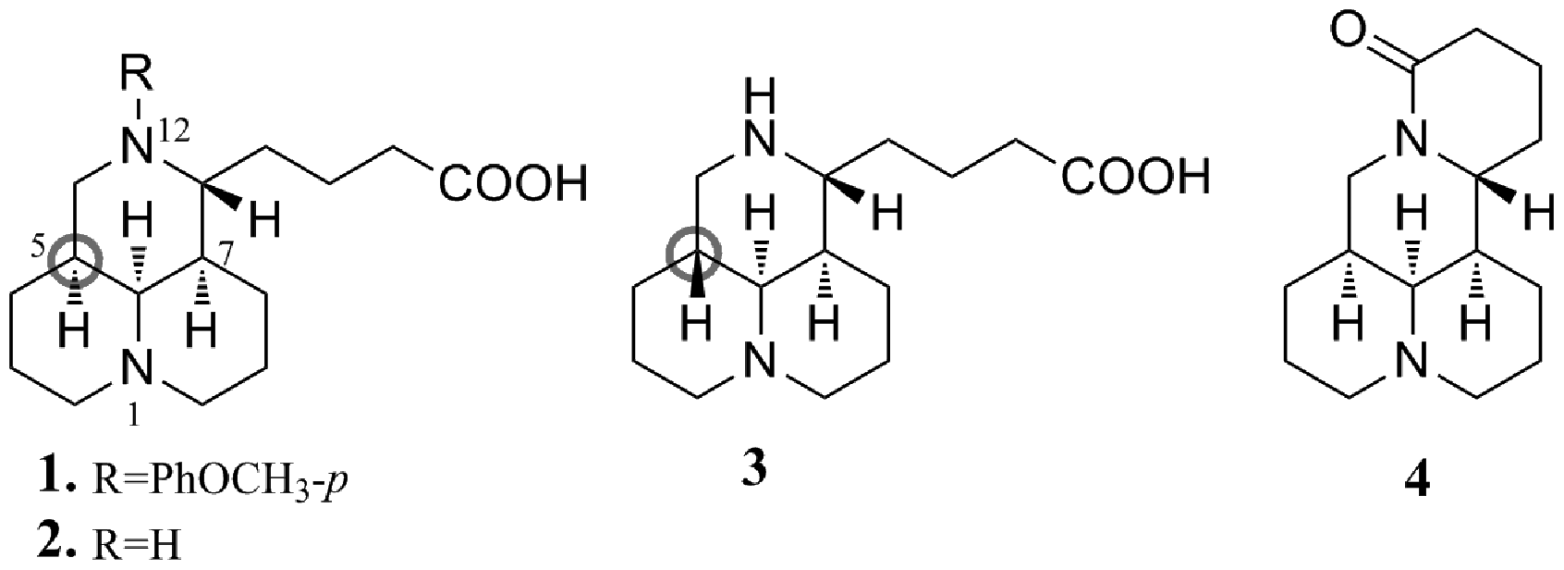
Chemical structures of compunds 1–4.

It was reported that host heat-stress cognate 70 (Hsc70) protein played an important role in the HCV replication cycle. We have demonstrated that host Hsc70 was a new drug target and mechanism against HCV [12]. Using Hsc70 as a target for HCV inhibition, we found that 12-*N*-*p*-methoxybenzyl matrinic acid (**1**, Figure 1) synthesized in our laboratory showed an anti-HCV activity [13]. It significantly down-regulates host Hsc70 expression at the post transcriptional level through destabilizing Hsc70 mRNA [13]. Its mode of action is distinctly different from that of the current anti-HCV drugs such as telaprevir and boceprevir [14], [15]. As this target is not a viral enzyme, antiviral agents acting through this mechanism might inhibit viral replication with no or decreased chance of causing drug-resistant mutations. This unique action mode and special scaffold of compound **1** strongly provoked our curiosity to explore the structure–activity relationship (SAR), with a goal of discovering novel anti-HCV agents.

Our previous SAR results [16], [17] indicated that (i) carboxyl group in **1** was considered of significant importance in down-regulating Hsc70 expression; (ii) substituted benzyl might significantly enhance the activity. As the inhibition rate of intracellular HCV replication was basically consistent with activity in Hsc70 down-regulation [13], [16], [17], SAR analysis for the inhibiting HCV replication was conducted with **1** as the lead in the present study. We retained the butyric acid chain, and focused the SAR study on the influence of the substituents on the phenyl ring including electron-withdrawing and electron-donating, and the effect of (*S*)- or (*R*)-configuration of the chiral carbon at the 5-position, respectively. On the basis of this strategy, a series of new *N*-substituted benzyl matrinic acid (**2**, 5*S*-configuration, Figure 1) and sophoridinic acid (**3**, 5*R*-configuration, Figure 1) derivatives [18], [19] was designed and synthesized. Herein, we describe the synthesis, *in vitro* anti-HCV evaluation, SAR analysis, *in vivo* toxicity, as well as pharmacokinetics of this kind of compounds.

## Results and Discussion

### Chemical Synthesis

**Figure 2:**
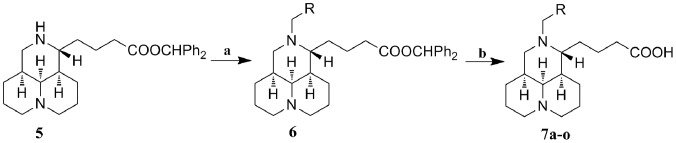
Reagents and conditions. : (a) RCH_2_X, K_2_CO_3_/CH_2_Cl_2_, rt, 9−24 h; (b) 6 M HCl, reflux, 1 h; then 3 M KOH.

**Figure 3:**
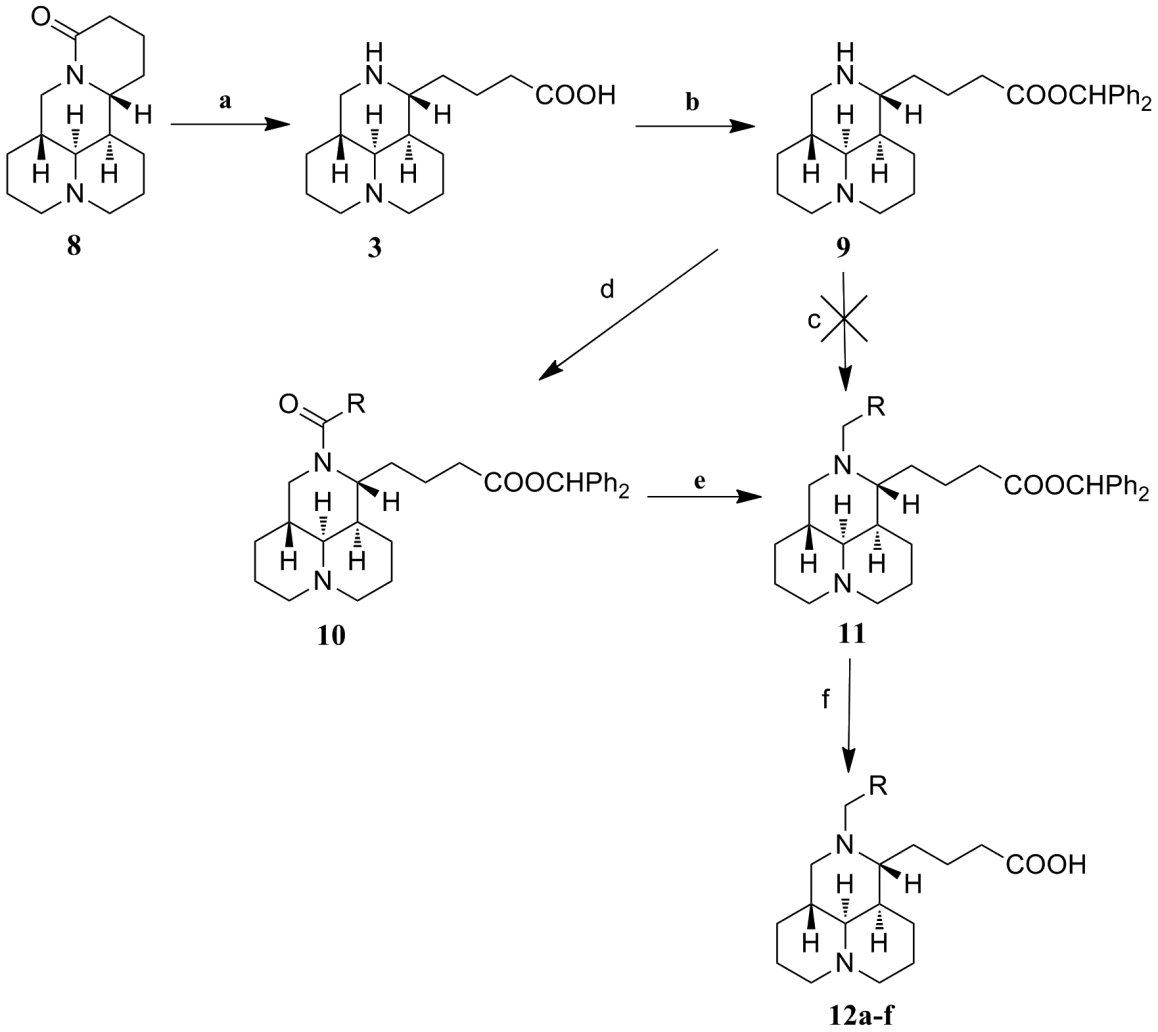
Reagents and conditions. : (a) KOH/H_2_O, reflux, 9 h; then 3 M HCl; (b) diphenyldiazomethane, MeOH/petroleum ether, overnight; (c) RCH_2_X, K_2_CO_3_/CH_2_Cl_2_, rt, 9−24 h; (d) RCOX, K_2_CO_3_/CH_2_Cl_2_, rt, 6−8 h; (e) BMS, THF, rt, 6 h; (f) 6 M HCl, reflux, 0.5 h, then 3 M KOH.

Twenty-one new target compounds were grouped into matrinic core and sophoridinic core and then synthesized as described in Figure 2 and Figure 3, respectively. The key intermediate **6** was prepared with commercially available matrine (**4**) as the starting material, using a three-step sequence including hydrolysis, carboxyl protection *via* diphenyldiazomethane [20], [21] and 12-*N*-alkylation with the methods reported previously [17]. Using hydrochloric acid as a de-protective reagent [22], the desired products **7a**–**o** were obtained in 6 M HCl at refluxing temperature for 0.5–1 h with yields of 50–60%.

The second synthetic route used commercially available sophoridine (**8**) as the starting material. The synthetic strategies shown in Figure 3 illustrated our efforts on obtaining the key intermediate **11** through the reduction reaction of acyl group on the 12-nitrogen atom, rather than 12-*N*-alkylation reaction. Intermediate **9** was synthesized with the methods similar to that of **5** [17], and then converted into the corresponding 12-*N*-acyl sophoridinic acids (**10**) in CH_2_Cl_2_ in the presence of K_2_CO_3_. Intermediate **11** was acquired through a selective reduction of **10**, in which borane dimethyl sulfide (BMS) [23], [24] was used as the reductive agent and THF as the solvent. Finally, all of the final products in series **7** and **12** were purified with silica gel column chromatography using CH_2_Cl_2_/MeOH as gradient eluent.

**Figure 4:**
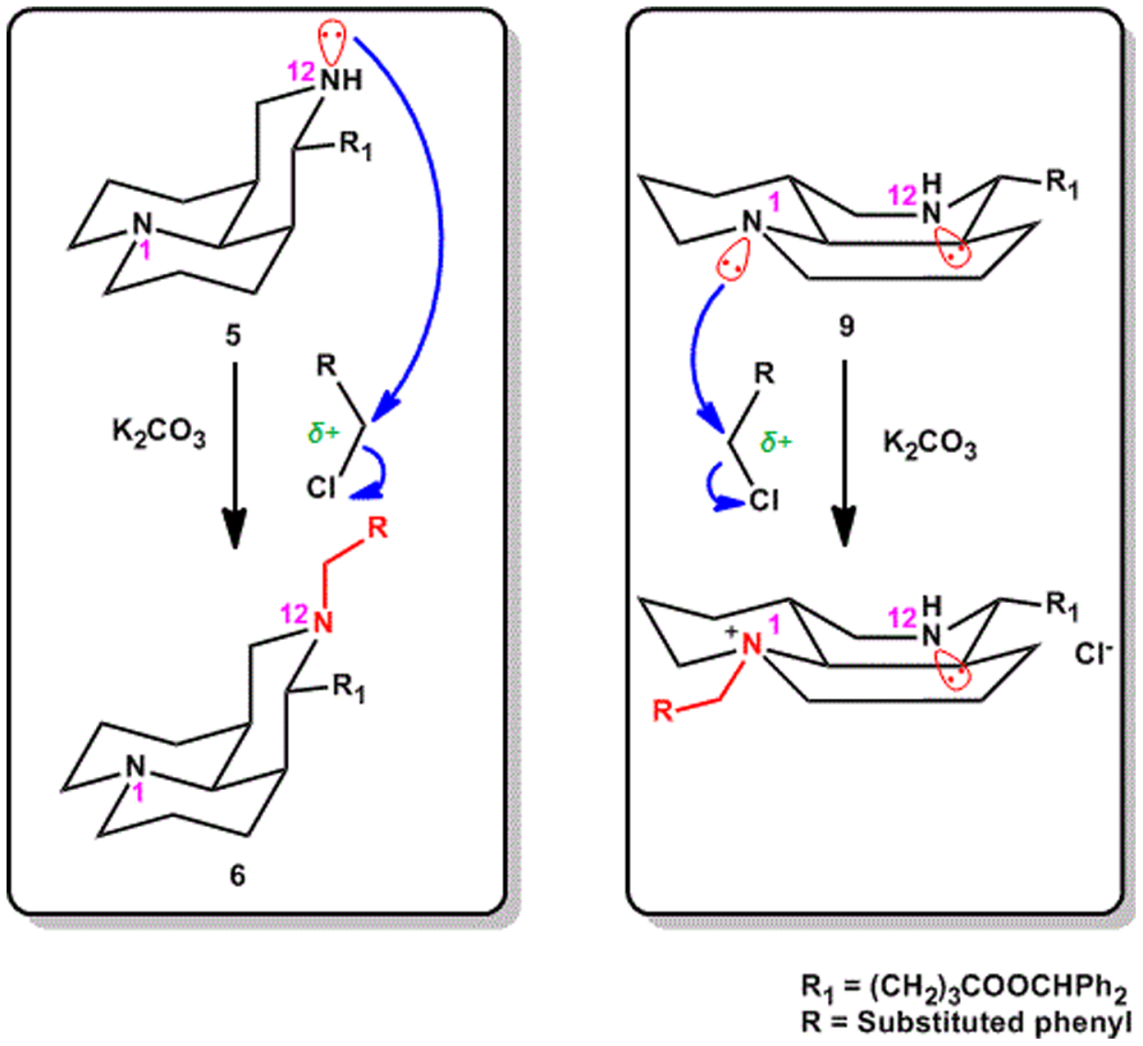
The conformation analysis of the matrinic series (left) and its isomeric sophoridinic series (right), to deduce the feasiblity of the alkylation reaction on the 12-nitrogen atom in sophoridinic series.

In the 12-*N*-alkylation in **5** series, the *N*-alkyl matrinic acid **6** was obtained as expected, because the lone pair electron on the 12-nitrogen atom could easily attack at the carbonium ion in RX. As shown in Figure 4 (left), the conformation analysis of matrinic core [18], [19] could be a reasonable explanation for the *N*-alkylation at the 12-position. However, in the 12-*N*-alkylation of sopharidinic core in **9** series, the *N*-alkylation of **9** with RX took place just on the 1-*N*-nitrogen atom (Figure 4), owing to the steric hindrance at the 12-position in sophoridinic series.

### SAR Analysis for Anti-HCV Activity *in vitro*

All of the synthesized compounds were examined for their anti-HCV activity and cytotoxicity in Huh 7.5 cells using specific real-time RT-PCR assay, as described in our previous publication [13]. Anti-HCV activity was evaluated by measuring both EC_50_ (for anti-HCV activity) and CC_50_ (for cytotoxicity) values. As a key indication, the selectivity index (SI) was calculated as a ratio of CC_50_ to EC_50_. Anti-HCV activity of the study compound was estimated by combining its EC_50_ value with SI. Structures of 21 *N*-benzyl matrinic/sophoridinic acid analogues and their anti-HCV effect were shown in Table 1.

**Table 1:** Structures and Anti-HCV Activity in Huh7.5 Cells of the Target Compounds.

Compd	12-R^a^	EC_50_ (µM)	CC_50_ (µM)	SI^b^
**1**	PhOCH_3_-*p*	118.5	>2590	>21.9
**7a**	PhCH_3_-*o*	203.3	1130	5.6
**7b**	PhCH_3_-*m*	265.9	2392	9.0
**7c**	PhCH_3_-*p*	51.1	>2702	>52.9
**7d**	PhF-*o*	87.4	1660	19.0
**7e**	PhF-*m*	168.2	1411	8.4
**7f**	PhF-*p*	176.5	>2673	>15.1
**7g**	PhCl-*o*	176.3	1290	7.3
**7h**	PhCl-*m*	88.5	>2557	>28.9
**7i**	PhCl-*p*	135.7	2499	18.4
**7j**	PhCl_2_-*3,4*	268.3	1006	3.7
**7k**	PhCl_2_-*2,4*	460.0	577.7	1.3
**7l**	PhBr-*p*	118.9	1230	10.3
**7m**	PhCH = CH_2_-*p*	4.70	184.2	39.2
**7n**	PhOCF_3_-*p*	88.5	1376	15.5
**7o**	C_10_H_7_	>821.0	1304	<1.6
**12a**	PhOCH_3_-*p*	57.5	1065	18.5
**12b**	PhCH_3_-*m*	453.3	1325	2.9
**12c**	PhCH_3_-*p*	503.2	2202	4.4
**12d**	PhF-*o*	>890.1	1547	<1.7
**12e**	PhF-*p*	107.5	1119	10.4
**12f**	PhOCF_3_-*p*	11.1	140.8	12.7
INF-α		>60 U/mL	0.35 U/mL	>171

a **7a**–**o**: 5*S*-configuration; **12a**–**f**: 5*R*-configuration.

b Selectivity index (SI) value equaled to CC_50_/EC_50_.

**Figure 5:**
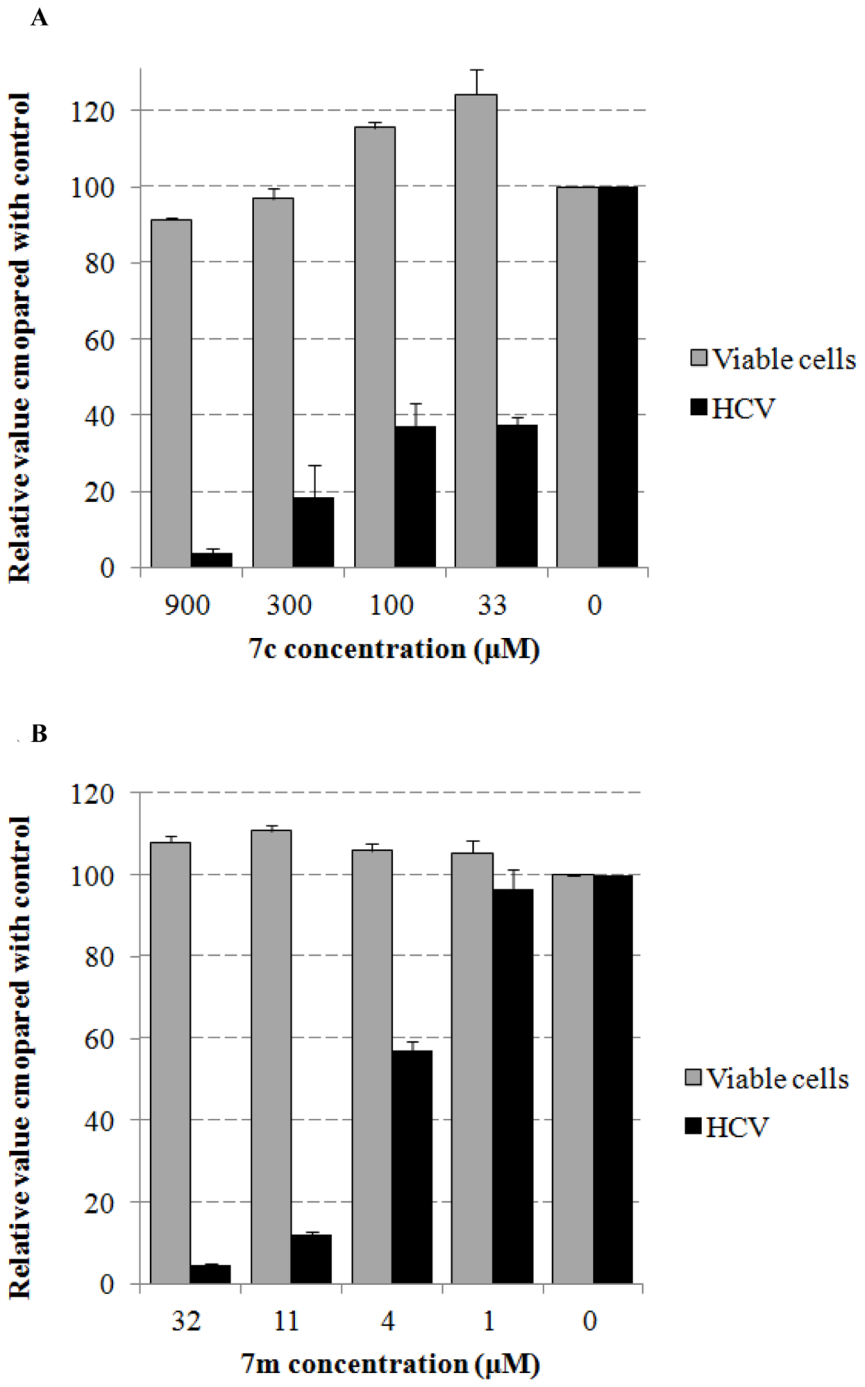
The dose-dependency of anti-HCV effect of both compounds 7c (A) and 7m (B).

SAR analysis was first focused on the influences of the substituents on the phenyl ring in **1**. Replacement of *p*-methoxy with *o*-, *m*- or *p*-methyl respectively gave compounds **7a**–**c**. Compound **7c** possessing *p*-methylbenzyl showed a 2-fold improvement in anti-HCV activity as compared to **1**. Similarly, attachment of *o*-, *m*- or *p*-fluoro at the phenyl ring resulted in compounds **7d**–**f**. All of them exhibited a moderate activity with EC_50_ between 87–176 µM, similar to or less than that of **1** (EC_50_ = 118 µM). Mono or di-chloro atom(s) was added at the aromatic ring respectively, by which five compounds (**7g**–**k**) were generated and tested. Compound **7h** bearing *m*-chlorobenzyl had slight improvement in comparison to the lead. Introduction of a *p*-bromo led to analogue **7l**, which showed a reasonable activity (EC_50_ = 119 µM) similar to that of **1**. The electron-withdrawing groups vinyl and OCF_3_ were attached to the phenyl ring, with which compounds **7m**–**n** were produced. The highest anti-HCV activity was seen in **7m** in this series (EC_50_ 4.70 µM). Compound **7o** possessing a naphthylmethylene lost the activity completely. The results suggested that introduction of a substituent, either electron-withdrawing (**7m**) or electron-donating (**7c**), to the phenyl ring could significantly enhance the anti-HCV activity. The dose-response curves of compounds **7c** and **7m** for anti-HCV effect were shown in Figure 5.

Next, the SAR analysis was moved on the effect of (*S*)- or (*R*)-configuration of the asymmetric center at the 5-position in **1**, in which six new derivatives of *N*-benzyl sophoridinic acid (**12a**–**f**) were prepared and tested. The results showed that compounds **12b–d** decreased their inhibition on HCV partially or completely, as compared to the corresponding matrinic acids (**7b**–**d**). Compounds **12a**, **12e**–**f** exhibited a moderate anti-HCV activity with SI values between 10.4 and 18.5, less than that of the corresponding matrinic acids (**1**, **7f**, **7n**) with SI ranges of 15.1 to 21.9. It appeared that the matrinic scaffold or 5*S*-configuration might play an important role in the antiviral activity against HCV.

### Anti-HCV Effect and Mode of Action

**Figure 6:**
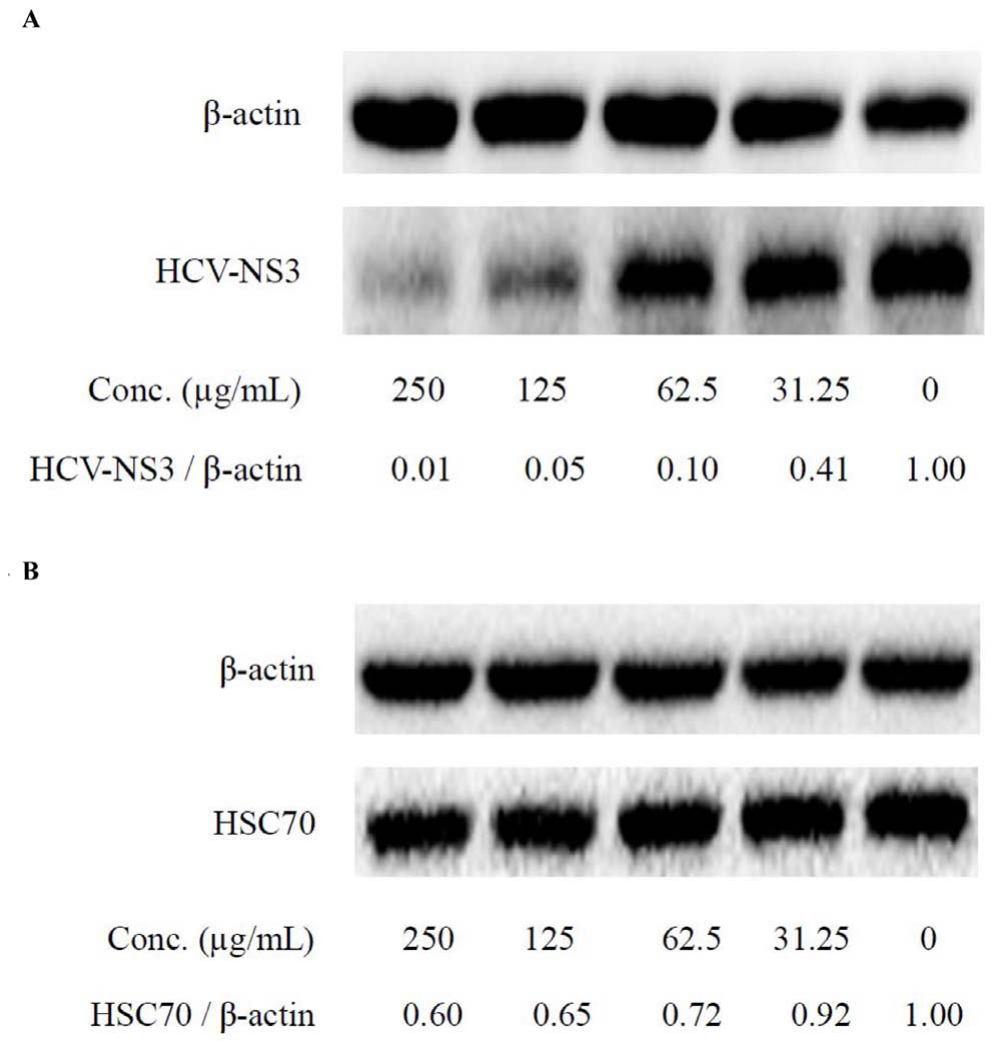
The intracellular HCV-NS3 (A) as well as Hsc70 protein (B) decreased dose-dependently in the Huh7.5 cells untreated or treated with 7c (31.25, 62.5, 125 and 250 µg/mL, respectively) for 72 h.

**Figure 7:**
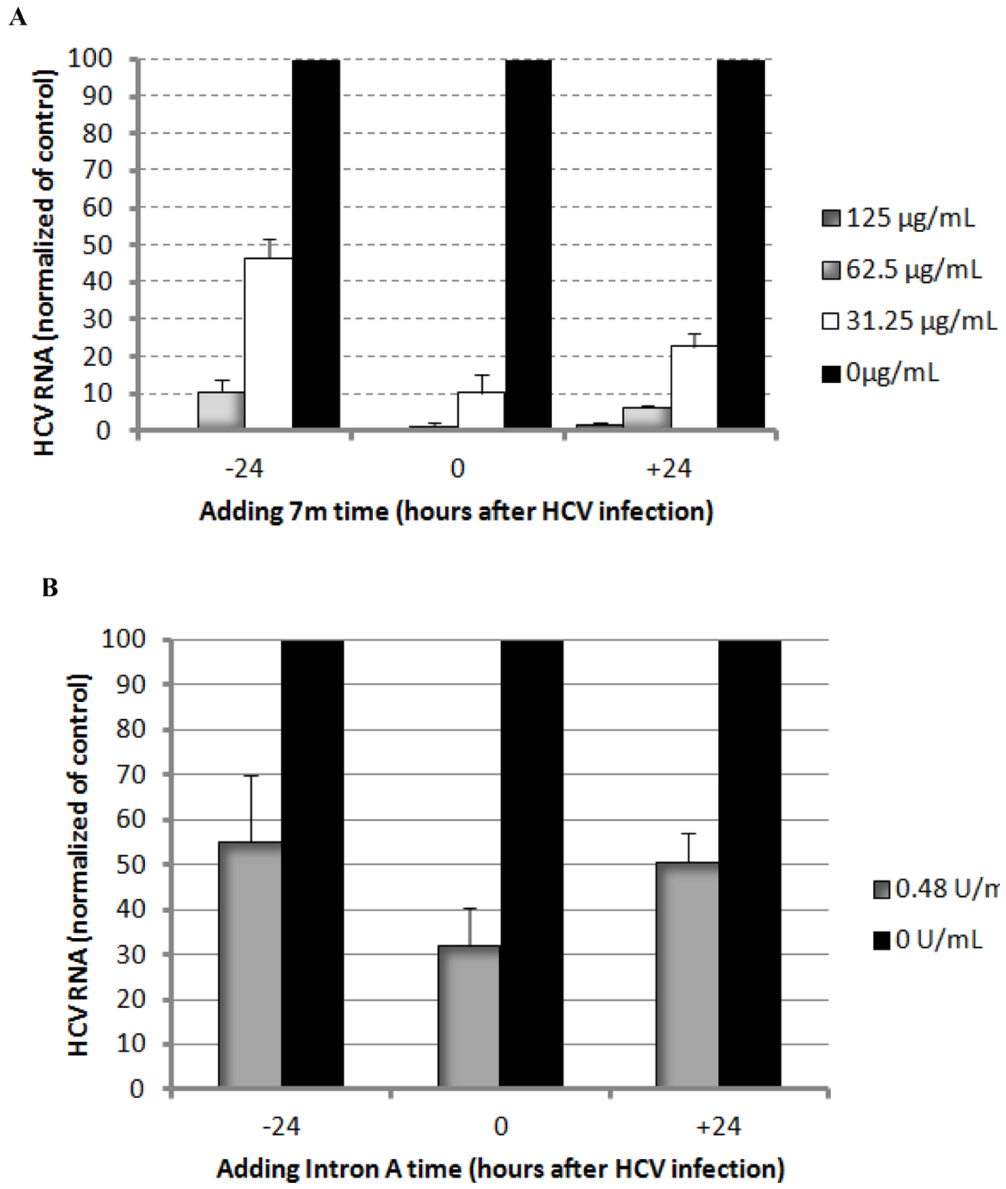
Therapeutic efficacy of 7m (A) and positive control Intron A (B) before, at and after infection in the Huh7.5 cells.

Since compound **7c** exhibited the most potent effect against HCV with SI of 53, it was selected to verify its anti-HCV effect at protein level in Huh7.5 cells. As shown in Figure 6A, compound **7c** treatment (62.5 µg/mL) significantly reduced HCV NS3 level, and the strongest anti-HCV effect was seen at the concentration of 250 µg/mL. To further confirm the mode of action for the activity against HCV, down-regulation of Hsc70 expression by **7c** was also examined by Western Blot. As shown in Figure 6B, compound **7c** afforded activity in down-regulating Hsc70 protein expression. As the anti-HCV activity of the compounds appeared over their effect on Hsc70, other mechanisms might be involved. Furthermore, therapeutic efficacy of **7m** before, at and after infection was measured as well and the results are shown in Figure 7. It appears that **7m** was effective before, at and after HCV infection, supporting its host environment-related action mode. The anti-HCV effect of compound **7m** was similar to that of the positive control Intron A (interferon a-2b), consistent with our previous report [13]. Compound **7c** exhibited an anti-HCV pattern close to that of **7m**.

**Figure 8:**
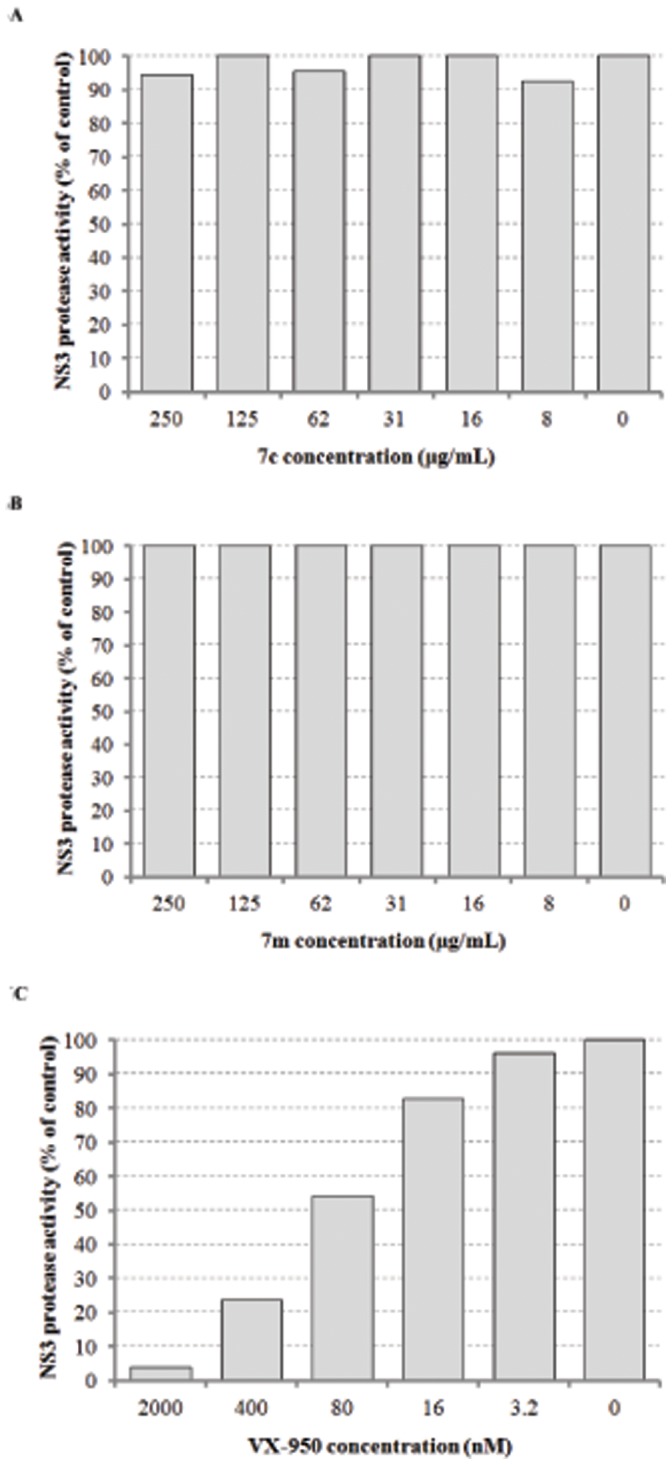
Effect of 7c and 7m on HCV RNA protease. Inhibitory activity on HCV RNA protease was examined with compounds **7c** (A) and **7m** (B) or the positive control VX-950 (C), respectively.

In addition, HCV NS3/4A protease activity (Michaelis constant Km = 0.60 µM) in the lysates was not altered after the treatment with compound **7c** or **7m** at the concentration of 250 µg/mL respectively (Figure 8A, 8B), while the positive control VX-950 showed a potent inhibitory activity on HCV RNA protease with IC_50_ of 98.8 nM (Figure 8C). Down-regulating stability of host Hsc70 mRNA seems to be the main mechanism of the compounds (Figure 6B) [13]. Therefore, we deduced that host Hsc70 might be at least one of the key drug targets for **7c** in its action against HCV. Compound **7c** working through down-regulating host Hsc70 expression might inhibit HCV replication with an advantage of no or decreased chance of inducing drug-resistant mutations.

### Pharmacokinetic and Safety Assessment of 7c and 7m

**Figure 9:**
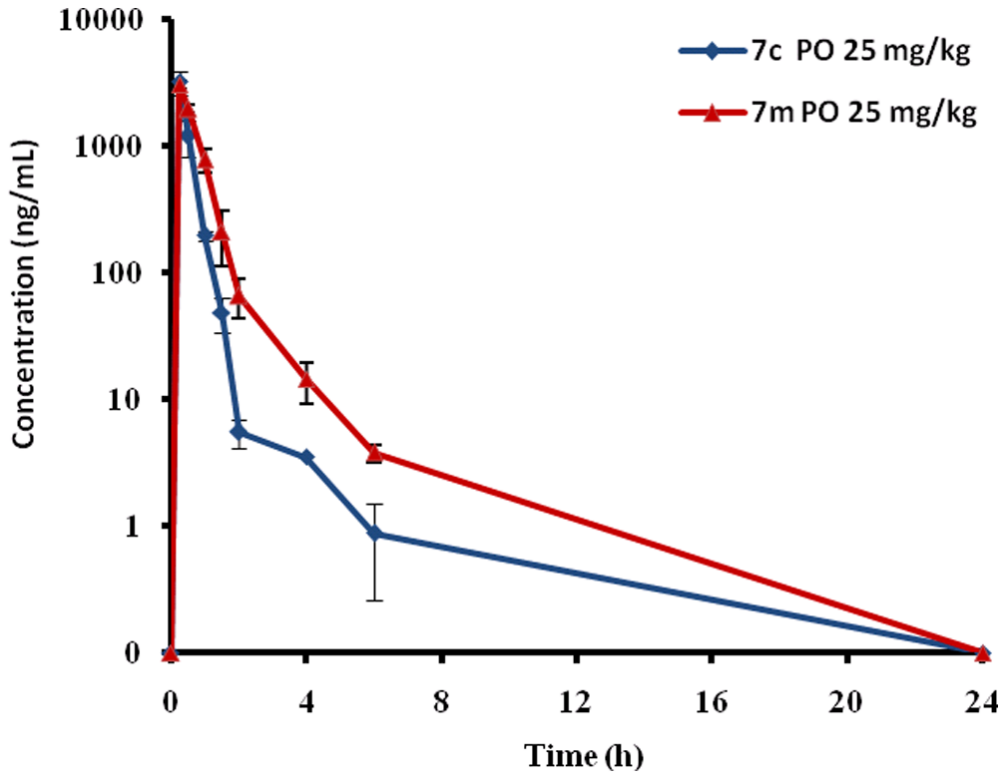
Mean plasma concentration-versus-time curve of 7c and 7m after oral administration at 25 mg/kg to mice (n = 3), respectively.

Among the aimed analogues, compounds **7c** and **7m** afforded an increased anti-HCV effect compared with **1**, both of them were chosen to evaluate their *in vivo* mice pharmacokinetic behavior in mice model. The study compounds were given to male ICR mice via oral (ig, 25 mg/kg) route. As indicated in Table 2, the absorption was rapid for both derivatives, and the maximum concentration (*C*_max_) in plasma after dosing was reached in 15 min and 30 min respectively. The *C*_max_ of **7m** was 23.2 µM, 1.8-fold of that of **7c** (*C*_max_ = 13.2 µM). The area under the curve (AUC) of **7m** (AUC = 25.9 µM·h) from 0 to 24 h was approximately 2-fold (Table 2 and Figure 9) higher than that of **7c** (AUC = 13.4 µM·h). The plasma concentration levels (*C*_max_ = 23.2 µM) of **7m** in mice were higher than its anti-HCV EC_50_ value *in vitro* (4.70 µM).

**Table 2:** Pharmacokinetic Parameters of Compounds **7c** and **7m** in Male ICR Mice.

Compd	T_max_(min)	C_max_(µM)	AUC _0−t_(µM·h)	MRT(min)	t_1/2_(min)
**7c**	15	13.2	13.4	100.8	90
**7m**	30	23.2	25.9	99	120.6

Single dose toxicity tests for **7c** and **7m** were carried out in mice as well. After **7c** or **7m** was given by intragastric administration (i.g) at a dose of 250, 500 or 1000 mg/kg, the mice were closely monitored for 7 days. No mouse died in the experiment duration, indicating that the LD_50_ value for **7c** or **7m** via oral route was over 1000 mg/kg. In addition, this treatment with **7c** or **7m** showed no effect on body weight of mice as well (data not shown). The results suggested that compounds **7c** and **7m** were considerably safe in vivo.

### Conclusions

In searching for novel anti-HCV agents that work through down-regulating host Hsc70 expression, a novel series of derivatives of *N*-benzyl matrinic or sophoridinic acids was synthesized and evaluated for their anti-HCV activities in Huh7.5 cells with **1** as the lead. SAR revealed that matrinic acid core was considered to be the optimal core structure for anti-HCV activity. Among the newly synthesized derivatives, compound **7c** exhibited a moderate inhibitory activity on HCV replication, with a novel mode of action distinctly different from the marketed anti-HCV drugs. In addition, compound **7c** showed a good PK profile and high safety in mice, indicating a druggable nature of the structure. Therefore, it was selected as a new mechanism anti-HCV candidate for further development, with a potential advantage of decreasing drug-resistant mutations in virus. In addition, combination of compound 7**c** with currently used anti-HCV drugs might provide a new regimen to improve therapeutic efficacy and reduce adverse effects.

## Methods

### Chemical Methods

#### Reagents and apparatus

Melting point (mp) was obtained with YRT-3 melting point apparatus and uncorrected. ^1^H-NMR and ^13^C-NMR spectra were performed on a Varian Inova 400 MHz spectrometer (Varian, San Francisco, CA) or 500 MHz spectrometer (AV500-III, Brvker, Swiss) in CD_3_OD, with Me_4_Si as internal standard. ESI high-resolution mass spectra (HRMS) were recorded on an AutospecUItima-TOF mass spectrometer (Micromass UK Ltd, Manchester, UK). Flash chromatography was performed on CombiflashRf 200 (Teledyne, Nebraska, USA), particle size 0.038 mm. All test compounds were confirmed to be ≥95% pure by HPLC.

#### General procedure for compound 7 and 12

Compound **4** or **8** (1 equiv) was added to a solution of KOH (6 equiv) in water. The reaction mixture was refluxed for 9 h, and then stirred at room temperature overnight. The reaction solution was cooled in ice-water bath, and acidified with HCl (3 M). The solvent was removed in vacuo and the residue was sufficiently dissolved in methanol to give a corresponding solution of crude **2** or **3**.

A mixture of diphenylmethanonehydrazone (1.5 equiv) and electrolytic MnO_2_ (1.5 equiv) in petroleum ether (boiling range 30–60°C) was refluxed for 4 h, to give a purple mixture of diphenyldiazomethane. The insoluble solid was filtered off and the clear filtrate was added into the solution of crude **2** or **3** (1 equiv) in methanol mentioned above. The reaction mixture was stirred at room temperature until the purple color disappeared, and then filtered. The resulting filtrate was evaporated under reduced pressure to dryness. The residue was washed with petroleum ether to afford crude compound **5** or **9** which was used for next step without further purification.

#### 12*-N*-2-Methylbenzyl matrinic acid (7a)

To the mixture of compound **5** and anhydrous K_2_CO_3_ (3 equiv) in CH_2_Cl_2_ was added 2-methylbenzyl bromide (1 equiv) dissolved in CH_2_Cl_2_. The reaction mixture was stirred at room temperature till the reaction was completed (checked by TLC), then filtered. The filtrate was evaporated *in vacuo* to give the crude product **6** as oily residue. Then compound **6** was dissolved in 6 M HCl, and the mixture was refluxed for 1 h, cooled, and 3 M KOH was added to neutralize the excessive HCl. The solution was extracted with ethyl acetate, and the aqueous layer was evaporated to dryness, and the residue was purified through flash chromatography over silica gel to give the title compound as a light brown solid. Yield: 35%, m.p 111−113°C. ^1^H NMR (CD_3_OD, 400 MHz): δ 7.36 (d, *J* = 6 Hz, 1 H), 7.17 (m, 3 H), 4.37 (d, *J* = 13.2 Hz, 1 H), 3.67 (d, *J* = 13.2 Hz, 1 H), 3.18−3.05 (m, 3 H), 2.86 (s, 2 H), 2.60−2.50 (m, 3 H), 2.37 (s, 3 H), 2.30−2.25 (m, 2 H), 2.10−1.54 (m, 13 H), 1.40 (d, *J* = 10.4 Hz, 1 H); ^13^C NMR (CD_3_OD, 400 MHz): δ 178.6, 138.9, 134.6, 131.9, 131.6, 129.4, 127.4, 64.7, 60.4, 57.3 (3), 54.5, 50.9, 37.9, 35.4, 33.3, 28.9, 27.2, 26.6, 20.7 (2), 19.8; HRMS: calculated C_23_H_34_N_2_O_2_ (M+H)^+^371.2699, found 371.2686.

#### 12-*N*-3-Methylbenzyl matrinic acid (7b)

The title compound was obtained from **5** and 3-methylbenzyl bromide with a procedure similar to that of **7a**. Yield: 38%. Light yellow solid, m.p 103−105°C. ^1^H NMR (CD_3_OD, 500 MHz): δ 7.39 (s, 1 H), 7.34−7.27 (m, 3 H), 4.22−4.12 (m, 2 H), 3.84 (t, *J* = 13.5 Hz, 1 H), 3.59 (s, 1 H), 3.42–3.35 (m, 2 H), 3.04−2.93 (m, 3 H), 2.45−2.41 (m, 4 H), 2.35 (s, 3 H), 2.15−1.71 (m, 12 H), 1.56 (d, *J* = 13.5 Hz, 1 H); ^13^C NMR (CD_3_OD, 400 MHz): δ 176.6, 140.7, 133.1, 132.0, 130.4, 130.4, 129.6, 62.6, 58.7, 56.5 (3), 53.6, 50.1, 37.4, 34.9, 33.3, 28.5, 25.4, 25.0, 21.3, 21.1, 19.5; HRMS: calculated C_23_H_34_N_2_O_2_ (M+H)^+^371.2699, found 371.2683.

#### 12-*N*-4-Methylbenzyl matrinic acid (7c)

The title compound was obtained from **5** and 4-methylbenzyl bromide with a procedure similar to that of **7a**. Yield: 34%. Light yellow solid, m.p 106−108°C. ^1^H NMR (CD_3_OD, 500 MHz): δ 7.43 (d, *J* = 8 Hz, 2 H), 7.27 (d, *J* = 8 Hz, 2 H), 4.19 (s, 1 H), 4.11 (s, 1 H), 3.83 (t, *J* = 13.5 Hz, 1 H), 3.59 (s, 1 H), 3.42−3.35 (m, 2 H), 3.04−2.93 (m, 3 H), 2.45−2.39 (m, 4 H), 2.33 (s, 3 H), 2.01−1.65 (m, 12 H), 1.55 (d, *J* = 14 Hz, 1 H);^ 13^C NMR (CD_3_OD, 400 MHz): δ 177.5, 140.4, 131.7 (2), 130.7 (3), 63.8, 60.8, 57.1 (3), 57.0, 50.9, 37.8, 34.2, 32.8, 28.3, 26.8, 26.4, 21.2, 20.6, 20.5; HRMS: calculated C_23_H_34_N_2_O_2_ (M+H)^+^371.2699, found 371.2695.

#### 12-*N*-2-Fluorobenzyl matrinic acid (7d)

The title compound was obtained from **5** and 2-fluorobenzyl chloride with a procedure similar to that of **7a**. Yield: 29%. Light yellow solid, m.p 90−93°C. ^1^H NMR (CD_3_OD, 500 MHz): δ 7.58 (t, *J* = 5.8 Hz, 1H), 7.45 (m, 1 H), 7.24 (t, *J* = 6 Hz, 1 H), 7.18 (t, *J* = 7.4 Hz, 1 H), 4.73 (d, *J* = 10.4 Hz, 1 H), 4.08 (d, *J* = 10.4 Hz, 1 H), 3.85 (d, *J* = 6.8 Hz, 1 H), 3.55 (t, *J* = 9.8 Hz, 1 H), 3.47 (s, 1 H), 2.89−2.98 (m, 2 H), 2.41–2.39 (m, 3 H), 2.41–2.32 (m, 4 H), 2.11−1.67 (m, 11 H), 1.56 (d, *J* = 11.2 Hz, 1 H);^ 13^C NMR (CD_3_OD, 400 MHz): δ 176.7, 161.9, 134.5, 133.2, 126.2, 117.1, 116.9, 63.4, 61.3, 56.6 (3), 50.7, 37.5, 33.8, 32.2, 28.7, 25.9, 25.5, 21.0 (2), 19.8; HRMS: calculated C_22_H_31_FN_2_O_2_ (M+H)^+^375.2448, found 375.2434.

#### 12-*N*-3-Fluorobenzyl matrinic acid (7e)

The title compound was obtained from **5** and 3-fluorobenzyl chloride with a procedure similar to that of **7a**. Yield: 33%. Light brown solid, m.p 81−83°C. ^1^H NMR (CD_3_OD, 400 MHz): δ 7.48−7.42 (m, 1 H), 7.38−7.35 (m, 2 H), 7.19−7.14 (m, 1 H), 4.92 (d, *J* = 12.4 Hz, 1 H), 4.09−4.02 (m, 2 H), 3.69 (t, *J* = 13.2 Hz, 1 H), 3.55 (m, 1 H), 3.40−3.33 (m, 2 H), 3.03−2.89 (m, 3 H), 2.44−2.37 (m, 4 H), 2.14−1.67 (m, 11 H), 1.54 (d, *J* = 14 Hz, 1 H); ^13^C NMR (CD_3_OD, 400 MHz): δ 176.8, 163.2, 132.3, 128.3, 119.2, 117.9, 117.7, 62.9, 61.6, 56.5 (3), 50.8, 37.4, 33.4, 32.1, 28.5, 25.6, 25.2, 20.9, 19.6 (2); HRMS: calculated C_22_H_31_FN_2_O_2_ (M+H)^+^375.2448, found 375.2436.

#### 12-*N*-4-Fluorobenzyl matrinic acid (7f)

The title compound was obtained from **5** and 4-fluorobenzyl chloride with a procedure similar to that of **7a**. Yield: 35%. Grey solid, m.p 85−87°C. ^1^H NMR (CD_3_OD, 400 MHz): δ 7.43 (dd, *J* = 5.4, 8.2 Hz, 2 H), 7.06 (t, *J* = 8.6 Hz, 2 H), 4.33 (d, *J* = 13.2 Hz, 1 H), 3.61 (d, *J* = 13.2 Hz, 1 H), 3.22−3.10 (m, 2 H), 3.00 (s, 1 H), 2.91 (t, *J* = 12.8 Hz, 1 H), 2.67−2.55 (m, 3 H), 2.32−2.27 (m, 2 H), 2.17−1.97 (m, 3 H), 1.87−1.53 (m, 11 H), 1.43 (d, *J* = 13.2 Hz, 1 H);^ 13^C NMR (CD_3_OD, 400 MHz): δ 179.5, 163.0, 133.1, 116.8 (2), 116.6 (2), 64.6, 59.5, 57.3, 57.2 (2), 50.7, 37.6, 35.8, 32.7, 28.6, 27.3, 26.8, 21.0, 20.8, 20.7; HRMS: calculated C_22_H_31_FN_2_O_2_ (M+H)^+^375.2448, found 375.2464.

#### 12-*N*-2-Chlorobenzyl matrinic acid (7g)

The title compound was obtained from **5** and 2-chlorobenzyl chloride with a procedure similar to that of **7a**. Yield: 32%. Light yellow solid, m.p 140−143°C. ^1^H NMR (CD_3_OD, 400 MHz): δ 7.68 (d, *J* = 6 Hz, 1 H), 7.54 (d, *J* = 7.6 Hz, 1 H), 7.48−7.40 (m, 2 H), 4.44 (s, 1 H), 3.56 (s, 1 H), 3.43−3.36 (m, 2 H), 3.07−2.92 (m, 4 H), 2.45−2.35 (m, 4 H), 2.16−1.70 (m, 13 H), 1.60 (d, *J* = 13.6 Hz, 1 H); ^13^C NMR (CD_3_OD, 400 MHz): δ 176.6, 137.1, 135.4, 133.2, 131.6 (2), 129.1, 62.6, 61.3, 56.4 (3), 50.6, 37.3, 33.5, 32.1, 28.8, 25.5, 25.1, 21.5, 19.6 (2); HRMS: calculated C_22_H_31_ClN_2_O_2_ (M+H)^+^391.2152, found 391.2167.

#### 12-*N*-3-Chlorobenzyl matrinic acid (7h)

The title compound was obtained from **5** and 3-chlorobenzyl chloride with a procedure similar to that of **7a**. Yield: 28%. Light brown solid, m.p 78−80°C. ^1^H NMR (CD_3_OD, 400 MHz): δ 7.58 (s, 1 H), 7.45−7.41 (m, 3 H), 4.68 (s, 1 H), 3.86 (s, 1 H), 3.46−3.37 (m, 2 H), 3.04−2.82 (m, 4 H), 2.43−2.40 (m, 2 H), 2.37−2.26 (m, 2 H), 1.92−1.55 (m, 14 H);^ 13^C NMR (CD_3_OD, 400 MHz): δ 176.9, 135.9, 131.6 (2), 131.4, 130.2, 130.0, 63.9, 60.6, 56.8 (3), 51.0, 37.6, 33.7, 32.7, 28.5, 26.0, 25.6, 20.5, 20.0, 19.9; HRMS: calculated C_22_H_31_ClN_2_O_2_ (M+H)^+^391.2152, found 391.2165.

#### 12-*N*-4-Chlorobenzyl matrinic acid (7i)

The title compound was obtained from **5** and 4-chlorobenzyl chloride with a procedure similar to that of **7a**. Yield: 30%. Light yellow solid, m.p 105−108°C. ^1^H NMR (CD_3_OD, 400 MHz): δ 7.43 (d, *J* = 8.4 Hz, 2 H), 7.37 (d, *J* = 8.4 Hz, 2 H), 4.54 (s, 1 H), 3.72 (s, 1 H), 3.42−3.31 (m, 3 H), 2.81−2.73 (m, 3 H), 2.35 (t, *J* = 6.4 Hz, 2 H), 2.23−2.15 (m, 2 H), 2.07−1.50 (m, 14 H);^ 13^C NMR (CD_3_OD, 400 MHz): δ 177.0, 135.8, 133.5, 133.0 (2), 130.1 (2), 64.2, 60.3, 56.8 (3), 50.9, 37.7, 33.9, 32.8, 28.5, 26.3, 25.8, 20.4, 20.1 (2); HRMS: calculated C_22_H_31_ClN_2_O_2_ (M+H)^+^391.2152, found 391.2167.

#### 12-*N*-3,4-Dichlorobenzyl matrinic acid (7j)

The title compound was obtained from **5** and 3, 4-dichlorobenzyl chloride with a procedure similar to that of **7a**. Yield: 22%. Light yellow solid, m.p 93−95°C. ^1^H NMR (CD_3_OD, 400 MHz): δ 7.50 (s, 1H), 7.40 (d, *J* = 8 Hz, 1H), 7.27 (d, *J* = 8 Hz, 1H), 4.13 (d, *J* = 13.6 Hz, 1 H), 3.27 (d, *J* = 13.6 Hz, 1 H), 3.07−2.98 (m, 2 H), 2.89 (d, *J* = 10.4 Hz, 1 H), 2.75 (s, 1 H), 2.65 (t, *J* = 12.4 Hz, 1 H), 2.47−2.36 (m, 3 H), 2.13 (t, *J* = 6 Hz, 2 H), 2.00−1.90 (m, 3 H), 1.75−1.37 (m, 11 H);^ 13^C NMR (CD_3_OD, 400 MHz): δ 180.8, 140.6, 133.3, 132.0 (2), 131.6, 130.0, 65.5, 58.1, 57.0 (2), 55.1, 51.2, 37.9, 37.3, 33.3, 29.2, 26.9, 26.3, 21.2, 20.6, 20.5; HRMS: calculated C_22_H_30_Cl_2_N_2_O_2_ (M+H)^+^425.1763, found 425.1770.

#### 12-*N*-2,4-Dichlorobenzyl matrinic acid (7k)

The title compound was obtained from **5** and 2, 4-dichlorobenzyl chloride with a procedure similar to that of **7a**. Yield: 30%. Light yellow solid, m.p 112−114°C. ^1^H NMR (CD_3_OD, 400 MHz): δ 7.68 (d, *J* = 8.4 Hz, 1 H), 7.56 (s, 1 H), 7.40 (d, *J* = 8.4 Hz, 1 H), 4.20 (s, 1 H), 3.85 (s, 1 H), 3.55 (s, 2 H), 3.40−3.33 (m, 2 H), 3.03−2.86 (m, 3 H), 2.38 (t, *J* = 6 Hz, 3 H), 2.11−1.56 (m, 14 H);^ 13^C NMR (CD_3_OD, 400 MHz): δ 176.7, 137.5, 136.1, 131.0 (2), 129.2 (2), 63.5, 61.8, 56.5 (3), 50.6, 37.6, 33.6, 32.3, 28.7, 25.7, 25.4, 21.1, 19.7 (2); HRMS: calculated C_22_H_30_Cl_2_N_2_O_2_ (M+H)^+^425.1763, found 425.1770.

#### 12-*N*-4-Bromobenzyl matrinic acid (7l)

The title compound was obtained from **5** and 4-bromobenzyl bromide with a procedure similar to that of **7a**. Yield: 27%. Light yellow solid, m.p 133−135°C. ^1^H NMR (CD_3_OD, 400 MHz): δ 7.49 (d, *J* = 8.4 Hz, 2 H), 7.32 (d, *J* = 8.4 Hz, 2 H), 4.24 (d, *J* = 12 Hz, 1 H), 3.60 (d, *J* = 12 Hz, 1 H), 3.10−3.02 (m, 3 H), 2.83 (m, 2 H), 2.61−2.51 (m, 3 H), 2.34−2.21 (m, 2 H), 2.13−2.11 (m, 1 H), 2.03−1.96 (m, 2 H), 1.84−1.43 (m, 11 H);^ 13^C NMR (CD_3_OD, 400 MHz): δ 180.7, 135.3, 133.1 (2), 132.8 (2), 123.4, 64.8, 59.1, 57.4, 57.3, 53.8, 50.7, 37.5, 36.7, 32.5, 28.8, 27.4, 26.9, 21.1, 21.0, 20.9; HRMS: calculated C_22_H_31_BrN_2_O_2_ (M+H)^+^435.1647, found 435.1647.

#### 12-*N*-4-Vinylbenzyl matrinic acid (7m)

The title compound was obtained from **5** and 3-vinylbenzyl chloride with a procedure similar to that of **7a**. Yield: 36%. Light brown solid, m.p 129°C. ^1^H NMR (CD_3_OD, 500 MHz): δ 7.43−7.31 (m, 4 H), 6.79−6.66 (m, 1 H), 5.76 (d, *J* = 17.6 Hz, 1 H), 5.29 (d, *J* = 10.8 Hz, 1 H), 4.38 (d, *J* = 13.2 Hz, 1 H), 3.34−3.25 (m, 1 H), 3.05−2.96 (m, 3 H), 2.80 (m, 1 H), 2.67−2.62 (m, 1 H), 2.50−2.43 (m, 2 H), 2.32−2.24 (m, 2 H), 2.16−2.06 (m, 2 H), 1.99−1.87 (m, 2 H), 1.80−1.41 (m, 11 H);^ 13^C NMR (CD_3_OD, 400 MHz): δ 179.2, 139.4, 137.5, 131.4, 127.7 (4), 114.9, 64.4, 59.7, 57.3, 57.2 (2), 50.8, 37.6, 35.6, 32.7, 28.6, 27.2, 26.7, 20.9, 20.7, 20.6; HRMS: calculated C_24_H_34_N_3_O_4_ (M+H)^+^383.2699, found 383.2686.

#### 12-*N*-4-Trifluoromethoxybenzyl matrinic acid (7n)

The title compound was obtained from **5** and 4-trifluoromethoxybenzyl chloride with a procedure similar to that of **7a**. Yield: 43%. Light yellow solid, m.p 81°C (decomp). ^1^H NMR (CD_3_OD, 400 MHz): δ 7.49 (d, *J = *12 Hz, 2 H), 7.24 (d, *J* = 12 Hz, 2 H), 4.28 (d, *J* = 12 Hz, 1 H), 3.63 (d, *J* = 12 Hz, 1 H), 3.12−3.04 (m, 3 H), 2.85−2.78 (m, 2 H), 2.59−2.50 (m, 3 H), 2.28−2.12 (m, 3 H), 2.04−1.96 (m, 2 H), 1.84−1.81 (m, 1 H), 1.71−1.52 (m, 9 H), 1.45−1.42 (m, 1 H);^ 13^C NMR (CD_3_OD, 400 MHz): δ 180.2, 150.2, 132.4 (2), 122.3 (4), 65.0, 58.9, 57.3, 57.1, 54.6, 50.9, 37.6, 36.4, 32.7, 28.8, 27.1, 26.6, 20.9 (2), 20.7; HRMS: calculated C_23_H_31_F_3_N_2_O_3_ (M+H)^+^441.2365, found 441.2338.

#### 12-*N*-1-Naphthylmethylenyl matrinic acid (7o)

The title compound was obtained from **5** and 1-chloromethyl naphthalene with a procedure similar to that of **7a**. Yield: 36%. Grey solid, m.p 180−182°C. ^1^H NMR (CD_3_OD, 400 MHz): δ 8.40 (d, *J* = 7.6 Hz, 1 H), 8.01 (d, *J* = 8 Hz, 1 H), 7.95 (d, *J* = 8 Hz, 1 H), 7.80 (d, *J* = 6.8 Hz, 1 H), 7.67 (t, *J* = 7.6 Hz, 1 H), 7.56 (t, *J* = 7.8 Hz, 2 H), 5.43 (s, 1 H), 4.40 (s, 1 H), 4.06 (s, 1 H), 3.54 (s, 1 H), 3.45–3.34 (m, 2 H), 3.03−2.89 (m, 2 H), 2.70−2.65 (m, 1 H), 2.55−1.60 (m, 16 H), 1.34 (d, *J* = 14 Hz, 1 H);^ 13^C NMR (CD_3_OD, 400 MHz): δ 176.7, 135.5, 133.8, 132.8, 132.3, 130.3, 128.8, 127.7, 126.6 (2), 124.6, 62.8, 62.6, 56.4 (3), 50.5, 37.6, 33.3, 32.4, 28.6, 25.4, 25.2, 21.1, 19.5, 19.4; HRMS: calculated C_26_H_34_N_2_O_2_ (M+H)^+^407.2700, found 407.2711.

#### 12-*N*-4-Methoxybenzyl sophoridinic acid (12a)

To a stirred mixture of **9** and anhydrous K_2_CO_3_ (3 equiv) in CH_2_Cl_2_ was added -methoxyl benzoyl chloride (1 equiv) in CH_2_Cl_2._ The reaction solution was stirred at room temperature till the reaction was completed (checked by TLC), and then filtered. The filtrate was evaporated in vacuo giving an oily residue **10**. Then **10** was dissolved in anhydrous THF and BMS (2 M, 1.7 eq.) was added. The reaction was be stirred at room temperature for 6 h. After the solvent was evaporated, 6 M HCl was added and the reaction was refluxed for 1 h, cooled, and 3 M KOH was added to neutralize the excessive HCl. The solvent was removed in vacuo, and the residue was purified through flash chromatography over silica gel affording the title compound as a light brown solid. Yield: 16%, m.p 99−101°C. ^1^H-NMR (CD_3_OD, 400 MHz): δ 7.17 (d, *J = *8.4 Hz, 2 H), 6.78 (d, *J* = 8.4 Hz, 2 H), 3.71 (s, 3 H), 3.37–3.55 (m, 4 H), 3.29–3.18 (m, 2 H), 2.98 (d, *J* = 12 Hz, 1 H), 2.57 (d, *J = *9.2 Hz, 1 H), 2.47 (dd, *J* = 3.2, 12 Hz, 1 H), 2.34–2.09 (m, 5 H), 2.00–1.35 (m, 12 H); ^13^C-NMR (CD_3_OD, 400 MHz): δ 182.2, 160.2, 132.6, 130.8 (2), 114.7 (2), 63.9, 61.7, 58.7, 55.7, 54.1, 51.7, 46.4, 38.7, 37.6, 29.5, 27.8, 25.3, 24.4, 24.1, 23.1, 19.3; HRMS: calculated C_23_H_34_N_2_O_3_ (M+H)^ +^387.2648, found 387.2637.

### 12-*N*-3-Methylbenzyl sophoridinic acid (12b)

The title compound was obtained from **9** and 3-methylbenzoyl chloride with a procedure similar to that of **12a**. Yield: 13%. Light brown solid, m.p 136°C (decomp). ^1^H-NMR (CD_3_OD, 400 MHz): δ 7.12–7.04 (m, 3 H), 6.97 (d, *J* = 7.8 Hz, 1 H), 3.60–3.51 (m, 2 H), 3.47–3.38 (m, 2 H), 2.99 (d, *J* = 10.0 Hz, 1 H), 2.61 (d, *J* = 10.0 Hz, 1 H), 2.46 (dd, *J* = 4.0, 11.6 Hz, 1 H), 2.35–2.21 (m, 2 H), 2.25 (s, 3 H), 2.21–2.08 (m, 3 H), 2.00–1.34 (m, 14 H); ^13^C-NMR (CD_3_OD, 400 MHz): δ 181.6, 140.7, 138.9, 130.3, 129.2, 128.7, 126.8, 64.2, 61.7, 59.4, 54.2, 51.7, 46.5, 38.5, 37.7, 29.5, 27.8, 25.2, 24.4, 24.1, 23.1, 21.5, 19.3; HRMS: calculated C_23_H_34_N_2_O_2_ (M+H)^ +^371.2699, found 371.2681.

#### 12-*N*-4-Methylbenzyl sophoridinic acid (12c)

The title compound was obtained from **9** and 4-methylbenzoyl chloride with a procedure similar to that of **12a**. Yield: 13%. Light brown solid, m.p 134°C (decomp).^ 1^H-NMR (CD_3_OD, 400 MHz): δ 7.17 (d, *J* = 8.0 Hz, 2 H), 7.10 (d, *J* = 8.0 Hz, 2 H), 3.52 (d, *J = *14.0 Hz, 1 H), 3.42 (d, *J* = 14.0 Hz, 1 H), 3.17 (t, *J* = 12.0 Hz, 2 H), 2.83–2.80 (m, 2 H), 2.67 (s, 1 H), 2.47–2.38 (m, 3 H), 2.27 (s, 3 H), 2.22 (d, *J = *7.2 Hz, 1 H), 2.16 (t, *J* = 6.4 Hz, 2 H), 2.02–1.87 (m, 3 H), 1.73–1.66 (m, 3 H), 1.51–1.43 (m, 5 H), 1.30–1.24 (m, 2 H); ^13^C-NMR (CD_3_OD, 400 MHz): δ 181.9, 137.6 (2), 129.9 (2), 129.6 (2), 64.0, 61.6, 59.1, 54.1, 51.7, 46.5, 38.5, 37.6, 29.5, 27.8, 25.3, 24.3, 24.1, 23.1, 21.1, 19.3; HRMS: calculated C_23_H_34_N_2_O_2_ (M+H)^ +^371.2699, found 371.2685.

#### 12-*N*-2-Flurobenzyl sophoridinic acid (12d)

The title compound was obtained from **9** and 2-flurobenzoyl chloride with a procedure similar to that of **12a**. Yield: 13%. Light brown solid, m.p 175°C (decomp). ^1^H-NMR (d^6^-DMSO, 400 MHz): δ 7.37 (t, *J* = 7.2 Hz, 1 H), 7.19–7.17 (q, *J* = 7.2 Hz, 1 H), 7.06 (q, *J = *7.62 Hz, 1 H), 6.96 (t, *J* = 7.2 Hz, 1 H), 3.64–3.42 (m, 4 H), 3.25–3.30 (m, 2 H), 3.17–3.14 (m, 1 H), 2.99–2.96 (m, 1 H), 2.64–2.61 (m, 1 H), 2.49–2.47 (m, 1 H), 2.34–1.80 (m, 10 H), 1.69–1.54 (m, 4 H), 1.42–1.37 (m, 2 H); HRMS: calculated C_22_H_31_FN_2_O_2_ (M+H)^ +^375.2448, found 375.2430.

#### 12-*N*-4-Flurobenzyl sophoridinic acid (12e)

The title compound was obtained from **9** and 4-flurobenzoyl chloride with a procedure similar to that of **12a**. Yield: 14%. Light brown solid, m.p 156°C (decomp). ^1^H-NMR (CD_3_OD, 400 MHz): δ 7.28 (dd, *J* = 5.6, 8.4 Hz, 2 H), 6.95 (t, *J = *8.4 Hz, 2 H), 3.59–3.28 (m, 5 H), 3.18 (d, *J* = 13.2 Hz, 1 H), 2.98 (d, *J = *10.0 Hz, 1 H), 2.58 (d, *J* = 10.0 Hz, 1 H), 2.44 (dd, *J* = 4.0, 11.2 Hz, 1 H), 2.35–2.29 (m, 1 H), 2.25–2.07 (m, 4 H), 2.01–1.34 (m, 12 H); ^13^C-NMR (CD_3_OD, 400 MHz): δ 182.1, 165.6, 136.5, 131.3 (2), 116.0, 115.7, 64.2, 61.6, 58.5, 54.1, 51.7, 46.4, 38.6, 37.5, 29.5, 27.8, 25.2, 24.4, 24.0, 23.1, 19.3; HRMS: calculated C_22_H_31_FN_2_O_2_ (M+H)^ +^375.2448, found 375.2458.

#### 12-*N*-4-Trifluoromethoxybenzyl sophoridinic acid (12f)

The title compound was obtained from **9** and 4-trifluromethoxybenzoyl chloride with a procedure similar to that of **12a**. Yield: 13%. Light brown solid, m.p 149°C (decomp). ^1^H-NMR (CD_3_OD, 400 MHz): δ 7.52 (d, *J* = 8.4 Hz, 1 H), 7.45 (d, *J* = 8.4 Hz, 1 H), 7.34 (d, *J* = 8.4 Hz, 2 H), 3.57–3.43 (m, 4 H), 3.07–2.97 (m, 2 H), 2.45–2.44 (m, 1 H), 1.97–1.20 (m, 19 H); ^13^C-NMR (CD_3_OD, 400 MHz): δ 172.2, 135.9, 130.3, 130.0 (2), 122.3 (3), 62.6, 61.9, 60.9, 55.7, 54.2, 54.1, 46.3, 39.0, 29.7, 27.1, 26.8, 24.0 (2), 23.3, 18.9; HRMS: calculated C_23_H_31_F_3_N_2_O_3_ (M+H)^ +^441.2365, found 441.2351.

### Biological Methods

#### Cell culture

Human liver cell line Huh7.5 cells (kindly provided by Vertex Pharmaceuticals, Inc., Boston, MA) were cultured in Dulbecco’s modified eagle medium (DMEM) supplumented with 10% inactivated fetal bovine serum and 1% penicillin-streptomycin (Invitrogen). Cells were digested with 0.05% trypsin- ethylene diamine tetraacetic acid (EDTA) and split twice a week.

#### Anti-HCV effect in vitro

Huh7.5 cells were seeded into 96-well or 6-well plates (Costar) at a density of 3×10^4^ cells/cm^2^. After 24 h incubation, the cells were infected with HCV viral stock (recombination virus strain J6/JFH/JC, 45 IU/cell) and simultaneously treated with the test compounds at various concentrations.

Concentrations or solvent as the control. The culture medium was removed after 72 h incubation, the intracellular total RNA (in 96-well plates) was extracted with RNeasy Mini Kit (Qiagen), and total intracellular proteins (in 6-well plates) were extracted with Cyto-Buster Protein Extraction Reagent added with 1 mM protease inhibitor cocktail. The intracellular HCV RNA was quantified with a real time one-step reverse-transcription polymerase chain reaction (RT-PCR). HCV core protein was detected with western blot (see below).

#### Cytotoxicity assay

Huh7.5 cells were seeded into 96-well plates (Costar) at a density of 3×10^4^ cells/cm^2^. After 24 h incubation. Fresh culture medium containing test compounds at various concentrations were added. 72 h later, Cytotoxicity was evaluated with 3-(4,5-Dimethylthiazol-2-yl)-2,5-diphenyltetrazolium bromide (MTT).

#### Western blot

The extracted total protein were denatured by adding 5× loading buffer (250 mM Tris-HCl, pH 6.8, 5% dithiothreitol, 10% SDS, 0.5% bromophenol blue, 50% glycerol), followed by boiling for 10 min. Proteins were analyzed with SDS-PAGE, then transfered onto nitrocellulose membranes by electroblotter. The membranes were blocked in 5% nonfat dry milk in TBS-T solusion (20 mM Tris, 150 mM NaCl, 0.1% Tween-20) for 1 h, and washed three times for 10 min each in the TBS-T. Membrane samples were probed with monoclonal antibody specific for protein of HCV core or Hsc70. As a control, monoclonal antibody to actin was used. After being washed with TBS-T, the membranes were respectively incubated with secondary antibody of goat anti-mouse (for HCV core), goat anti-rat (for Hsc70), or goat anti-rabbit (for actin) at RT for 1 h. Protein was detected using Immbilon Western Chemiluminescent HRP Substrate (Millipore Inc.) with Alpha Innotech Focus and Image Acquisition.

#### Safety evaluation in vivo

Animals were purchased from the Institute of Laboratory Animal Sciences, Chinese Academy of Medical Sciences and Peking Union Medical College, Beijing, China. All experimental procedures were approved by Biomedical Ethics Committee of Chinese Academy of Medical Sciences of animal use and protection. The mice were cared according to the institutional guidelines of the Chinese Academy of Medical Sciences. Male and female KunMing mice with weight of 22.0±1.0 g were fed with regular chow and housed in an air-conditioned room. The mice were randomly divided into five groups with 10 mice each (five male plus five female). The each group of mice were administrated orally **7c** and **7m** at 0 (saline as control), 500 or 1000 mg/kg, respectively. The test compounds were given in a single-dosing. Body weight as well as survival was closely monitored.

#### Pharmacokinetic studies

Male male ICR mice (20–40 g) were obtained from SLAC Laboratory Animal Inc. (Shanghai, China). Three male ICR mice were used in each study. Each of them was dosed with a tested compound at 25 mg/kg via oral administration. Nine blood samples were collected at 0, 0.25, 0.50, 1.0, 2.0, 4.0, 6.0 and 24 h and were immediately centrifuged to separate the plasma fractions. The separated plasma samples were stored at –20°C for analysis. Concentration-versus-time profiles were obtained for each analyte, and standard non-compartmental analysis was performed on the data using WinNonlin software, version 5.3, to recover the AUC and other non-compartmental parameters.
